# Predicting cancer risk based on family history

**DOI:** 10.7554/eLife.73380

**Published:** 2021-09-29

**Authors:** Michelle F Jacobs

**Affiliations:** 1 Internal Medicine, University of Michigan Ann Arbor United States

**Keywords:** mendelian modeling, cancer risk, statistical software, pedigree data, None

## Abstract

A new software package provides more accurate cancer risk prediction profiles and has the ability to integrate more genes and cancer types in the future.

**Related research article** Lee G, Liang JW, Zhang Q, Huang T, Choirat C, Parmigani G, Braun D. 2021. Multi-syndrome, multi-gene risk modeling for individuals with a family history of cancer with the novel R package PanelPRO. *eLife* **10**:e68699. doi: 10.7554/eLife.68699

Countless hours have been dedicated to researching cancer – how to prevent it, how to diagnose it early, and how to treat it. Yet, cancer remains a leading cause of death worldwide, accounting for almost 10 million fatalities in 2020.

Most cancers are caused by changes to genes that happen over a person’s lifetime. In rarer cases (about 5–10%), they start due to inherited genetic mutations that produce a predisposition to cancer. In these instances, also known as familial or hereditary cancer syndromes, the mutation is passed down from generation to generation. In these families, more members tend to develop cancers than expected – often of the same or related type – which can also start at a particularly early age.

It is important to identify people with such genetic mutations so that they – and any family members at higher risk – can undergo enhanced cancer screening. Family history can be a useful predictor of hereditary cancer risk (Blackford and Parmigiani, 2010). As such, risk prediction models that incorporate family history to estimate a person’s chance of having a mutation in a cancer predisposition gene or of developing cancer have been employed for many years (Chen et al., 2004).

Historically, such models have been particularly valuable for deciding who to offer genetic testing to when only few and often costly genetic tests were available (Fasching et al., 2007). In some cases, insurance companies require the risk estimate related to carrying a cancer-related genetic mutation to exceed a certain threshold (typically 5 or 10%) to reimburse the cost of a genetic test (Chen et al., 2006). As research advances, the number of genes available for cancer-related genetic testing has now reached over 100 and is likely to continue increasing. Nevertheless, older risk modeling programs generally include only a small number of genes in their predictions. Now, in eLife, Danielle Braun and colleagues – including Gavin Lee and Jane Liang as joint first authors – report on a new software package that has the capacity to evolve alongside advances in cancer research (Lee et al., 2021).

The researchers, who are based at ETH Zürich, EPFL, Harvard, the Dana-Farber Cancer Institute, and the Broad Institute, developed PanelPRO, a tool that uses evidence gathered from extensive literature reviews to model the complex interplay between genes and cancer risk. PanelPRO’s workflow consists of four main parts: input, preprocessing, algorithm, and output (Figure 1).

**Figure 1.**
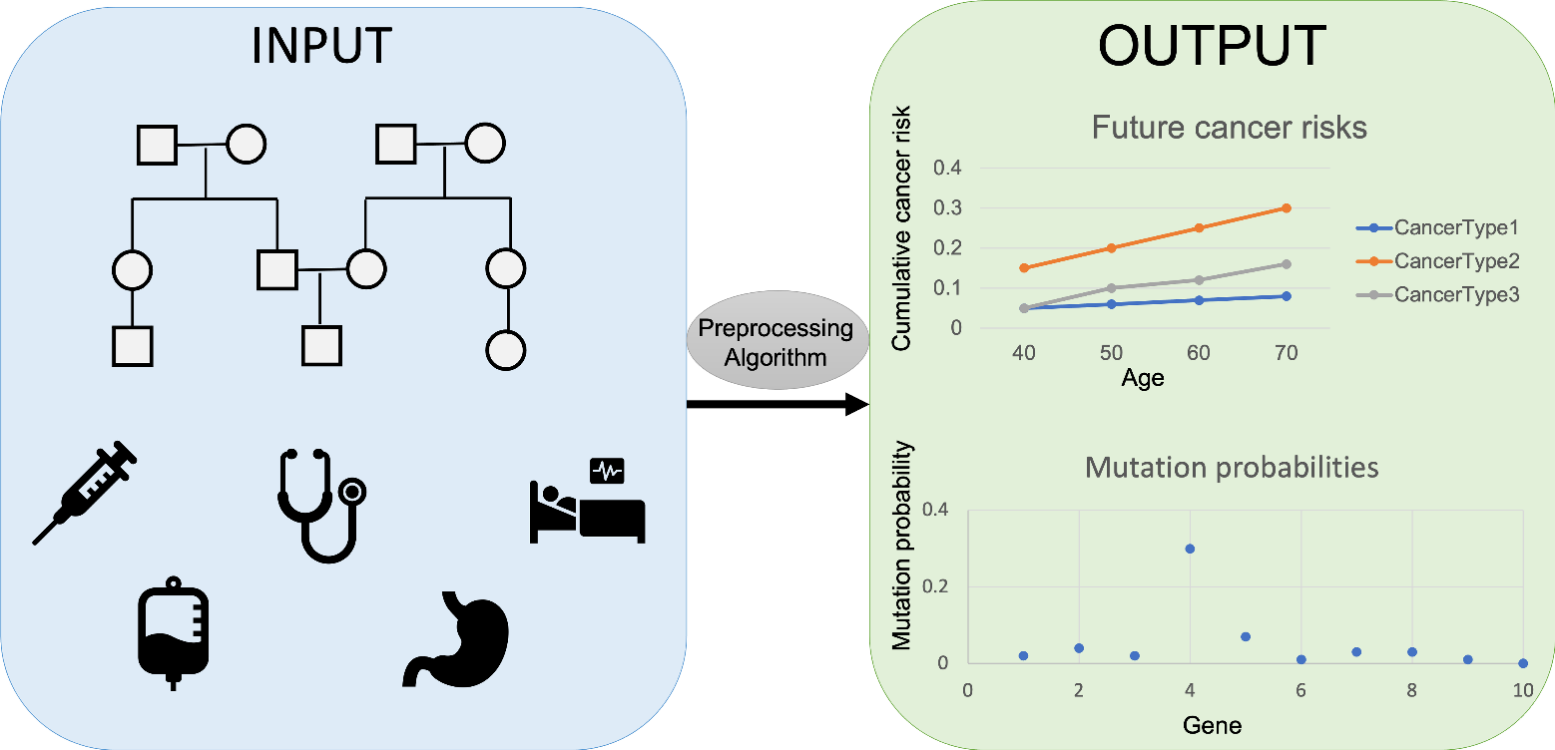
Workflow for PanelPRO. First, information on family history, including cancer diagnoses, age of relatives, and cancer risk factors is added into the risk modeling software PanelPRO (input, blue box on the left). Then, PanelPRO validates data formatting (preprocessing, grey oval), and analyses information about frequency and cancer risks for family cancer syndromes (algorithm, grey box) to estimate the likelihood of a person in a family having a mutation in a gene linked to an increased risk of cancer (output, green box on the right). Mutation probability and cumulative cancer risk are given as a probability between 0.0 (no risk) and 1.0 (100% risk).

The user first adds information about a history of cancers in a family – such as ages and cancer diagnoses – and other factors that might affect cancer risk. These include any risk-reducing surgeries in relatives, or tumors with biomarkers that might indicate a potential hereditary cause of their cancer. The software then adds information on the frequency of different hereditary cancer syndromes and assesses their associated cancer risks. PanelPRO can currently accommodate 18 types of cancer and generate predictions of probable mutations for 24 genes, but its code allows for the addition of new cancers or cancer-related genes that may be identified in the future.

During the preprocessing stage, the software verifies the input for any missing information and data, and also for any family relationships not supported by the software, such as ‘double cousins’, which occur when two siblings have children with two siblings from another family. Messages, warnings, or errors may be given to the user if any issues are detected.

After the information has been checked and modified as needed, the model proceeds to the algorithm stage. To calculate the output, the algorithm uses probabilities based on the family history, the frequency of hereditary cancer syndromes in the population, and the cancer history that would be expected if a cancer syndrome were present. The program then estimates the likelihood of a person in a family to have a mutation in a gene linked to an increased risk of cancer. These calculations can also be easily run for other family members using the existing information. It also shows a personalized estimate of future cancer risks. Users can choose which cancer types and genes to display.

However, some outstanding issues remain. Misreported family history information, such as an inaccurate cancer diagnosis or unknown age of diagnosis, can significantly affect estimates, highlighting that accuracy of patient-reported information is key to producing correct estimates (Katki, 2006). While patients have been shown to generally provide exact information on cancer history for first-degree relatives, the accuracy of these reports decreases for more distant relations (Augustinsson et al., 2018; Murff et al., 2004).

Moreover, analyses with a similar risk modeling software have revealed that a strict adherence to a 10% risk threshold to qualify for a test for a probable mutation in the BRCA gene (which is linked to an increased risk of developing breast, ovarian, and other cancers) would miss around 25% of individuals carrying a mutation when compared to genetic testing outcomes (Varesco et al., 2013). This is likely because cancer risks associated with hereditary cancer syndromes are more variable than initially appreciated, and not all family histories may exhibit a predictable pattern of cancer, even when a mutation is present (Okur and Chung, 2017). This complicates risk assessments and argues against making decisions about genetic testing solely based on risk prediction models. Today, broader insurance coverage guidelines and lower costs for genetic tests have increased clinicians’ ability to order these tests, even if certain risk thresholds are not met based on family history.

Nevertheless, the higher number of genes and cancer types supported by PanelPRO compared to other risk models are impressive and its ability to incorporate new genes and cancer types as testing advances are key in this fast-paced, constantly advancing field.
